# Assessment of Knowledge Regarding Risk of Peri-Implantitis After Dental Implant Placement Among Dental Practitioners of Gujarat State: A Questionnaire Survey

**DOI:** 10.7759/cureus.102385

**Published:** 2026-01-27

**Authors:** Rutvi Savalia, Rishabh Trivedi, Manjari Khatri, Dhruti Pobaru, Aayushi Patel, Keya Pancholi

**Affiliations:** 1 Dentistry, University of Glasgow, Glasgow, GBR; 2 Periodontology, K. M. Shah Dental College and Hospital, Sumandeep Vidyapeeth Deemed to be University, Vadodara, IND; 3 Dentistry, Government Dental College and Hospital, Jamnagar, IND

**Keywords:** clinical awareness, dentists, implant complications, implant maintenance, peri-implant diseases, peri-implantitis, peri-implant mucositis

## Abstract

Aims and background

Although dental implants have transformed prosthetic dentistry, implant health is seriously threatened by peri-implantitis, an inflammatory disease that affects the surrounding tissues. In order to prevent, identify, and treat peri-implantitis, dentists are essential. The purpose of this cross-sectional survey was to assess Gujarat state dentists' knowledge, comprehension, and clinical practices with reference to peri-implantitis.

Materials and methods

A structured questionnaire of 15 questions was distributed to dentists of Gujarat state including the topics of demographic data, basic knowledge of implant dentistry, knowledge of implant-related complications, attitude towards diagnosis & management of Implant-related complications revealing that while most were familiar with the term "peri-implantitis," significant knowledge gaps were observed in key areas such as risk factor identification, diagnostic procedures, and evidence-based management strategies.

Results

Results indicate that while the majority of practitioners were familiar with the term "peri-implantitis," there were considerable gaps in diagnostic acumen, understanding of risk factors, and treatment protocols.

Conclusion

This study underscores the urgent need for continuing education programs and improved dissemination of evidence-based guidelines to mitigate peri-implant disease incidence.

Clinical significance

The need for early peri-implantitis diagnosis and intervention, standardized treatment protocols, patient-centred care, periodic maintenance, recall intervals, and advanced technology to prevent tissue destruction and implant failure should be emphasized.

## Introduction

Over the past 20 years, dentistry has grown rapidly and is now a crucial part of all-inclusive dental treatment plans. Dental implants are generally regarded as the gold standard for the rehabilitation of edentulous areas, with success rates often reported above 90% over a 10-year period (Buser et al., 2012) [1]. However, careful upkeep and early identification of peri-implant issues are necessary for their long-term stability.

Peri-implant mucositis and peri-implantitis are the two general groups into which peri-implant illnesses fall. Peri-implantitis entails gradual bone loss and is a more serious clinical concern than peri-implant mucositis, which is defined by reversible inflammation of the soft tissues surrounding an implant without bone loss (Schwarz et al., 2018) [2]. The 2017 World Workshop on the Classification of Periodontal and Peri-Implant Diseases and Conditions standardized diagnostic criteria and emphasized the need for longitudinal assessments.

Peri-implantitis has emerged as a prevalent and increasingly recognized complication associated with dental implants. The prevalence varies significantly across studies due to heterogeneity in diagnostic criteria and follow-up duration. A systematic review by Derks & Tomasi (2015) [3] reported peri-implantitis in approximately 10% of implants and 20% of patients. Recent meta-analyses have even suggested high prevalence rates at the patient level (Diaz et al., 2022) [4].

Dentists frequently place, restore, and maintain dental implants, particularly in semi-urban and urban Indian contexts where specialist services may not be readily accessible. Therefore, it becomes crucial for dentists to identify and treat peri-implant disorders. There are still extensive gaps in non-specialists' knowledge of peri-implant illness, despite this.

In addition to antibiotic therapy employing systemic and local delivery systems, clinical management strategies for bone disorders include non-surgical techniques such as mechanical debridement, air-abrasive systems, laser therapy, and antiseptic application. Surgical procedures such as guided bone regeneration and open flap debridement are necessary for advanced instances. Even with several approaches, results can vary, and total illness resolution is still difficult to achieve (Renvert & Polyzois, 2015) [5].

So, the aim of this study was to assess the level of knowledge, awareness, and clinical practices of dentists in Gujarat state regarding peri-implantitis, including its risk factors, diagnosis, prevention, and management. The objectives included: To assess dentists’ knowledge and understanding of peri-implant diseases, including peri-implant mucositis and peri-implantitis; To determine the level of awareness among dentists regarding the risk factors and diagnostic criteria associated with peri-implantitis; To evaluate clinical attitudes and preferred management strategies adopted by dentists for peri-implantitis; To identify perceived challenges and barriers faced by dentists in the diagnosis, treatment, and maintenance of peri-implant diseases; To assess dentists’ perceptions regarding the role of patient education, maintenance protocols, and advances in implant materials and techniques in preventing peri-implant diseases.

## Materials and methods

Dentists registered with the Dental Council of India (DCI) and currently practicing in Gujarat State were eligible to participate in the study. A structured, self-administered questionnaire was employed in this descriptive, cross-sectional study to evaluate the knowledge, awareness, and clinical attitudes of dental practitioners toward peri-implantitis and its risk factors. The survey instrument was disseminated digitally, ensuring wide accessibility and ease of response collection. The inclusion criteria were general dental practitioners and specialists currently practicing in Gujarat State, possessing the necessary clinical exposure to provide valid insights into implant-related experiences. The exclusion criteria consisted of dentists who were not actively practicing, including those exclusively engaged in academic, administrative, or non-clinical roles, as well as undergraduate students and interns, to ensure that responses reflected real-world clinical experience. 

The focus was restricted to clinically active dentists to ensure that the data reflected practical experience and real-world implant maintenance scenarios rather than purely theoretical understanding. A non-probabilistic convenience sampling technique was employed. The questionnaire was disseminated digitally using Google Forms through professional networks, dental associations, and social media platforms commonly used by dentists. This approach was chosen due to feasibility considerations and to maximize participation across different regions within the state. 

The sample size was calculated using a standard formula for cross-sectional surveys. Assuming a confidence level of 95%, a margin of error of 5%, and an anticipated response proportion of 50% (to ensure maximum sample size estimation in the absence of prior state-level data), the minimum required sample size was estimated to be approximately 384 participants. A total of 406 valid responses were ultimately included in the final analysis, thereby exceeding the calculated requirement.

Participants were recruited from multiple districts across Gujarat State, including urban and semi-urban regions. While the survey was not stratified district-wise, the digital distribution strategy allowed participation from dentists practicing in various geographic locations within the state, enhancing regional representation.

The questionnaire design was grounded in an extensive review of published literature, including similar surveys and clinical studies addressing peri-implant diseases, implant maintenance, and practitioner awareness levels. The questionnaire consisted of four main sections:

Basic information: Capturing years of professional experience and area of specialization.

Basic knowledge of implant dentistry: Assessing familiarity with implant components, peri-implant tissue anatomy, and common biological complications.

Knowledge of implant-related complications: Focusing on recognition, etiology, and differentiation between peri-implant mucositis and peri-implantitis.

Attitude towards diagnosis & management of Implant-related complications: Evaluating diagnostic preferences, treatment strategies, referral patterns, and perceived barriers to peri-implantitis management.

Most of the questions were multiple-choice or Likert scale-based, designed to yield quantifiable data suitable for descriptive statistical analysis. The anonymity of responses was maintained to encourage honest participation, and informed digital consent was obtained from all participants prior to data submission. Ethical clearance was obtained from the Institutional Ethics Committee before the commencement of the study.

The questionnaire used in the present study was self-designed, developed specifically to assess dentists’ knowledge, awareness, and clinical practices related to peri-implant diseases. The content was formulated after an extensive review of previously published surveys and literature on peri-implantitis and implant maintenance practices [3,4], to ensure relevance and alignment with existing research. However, the questionnaire was not directly adapted from any single previously validated instrument, as no standardized questionnaire addressing all intended domains was available.

Content and face validity were assessed by an expert panel comprising five subject specialists, including three periodontists and two prosthodontists, each with more than five years of clinical and academic experience in implant dentistry. The experts evaluated the questionnaire for clarity, relevance, comprehensiveness, and appropriateness of the items in relation to the study objectives. Based on their feedback, minor modifications were made to improve question wording, eliminate ambiguity, and ensure logical sequencing of items.

A pilot study was conducted among a small group of practicing dentists (n = 20) who were not included in the final analysis. The purpose of the pilot testing was to assess feasibility, clarity, time required to complete the questionnaire, and ease of understanding. Feedback from the pilot participants led to minor refinements in language and response options. No major structural changes were required, and the questionnaire was deemed suitable for final distribution.

Formal statistical reliability testing, such as Cronbach’s alpha, was not performed. This is acknowledged as a limitation of the study. The questionnaire primarily included factual knowledge items and descriptive attitude questions, and was intended for exploratory assessment rather than scale-based psychometric evaluation. Similar methodological approaches have been reported in comparable cross-sectional dental awareness surveys.

Data were collected over a two-month period, providing an adequate window for participant engagement and response validation. Data collected through Google Forms were downloaded and compiled in Microsoft Excel (Microsoft Corp., Redmond, WA, USA) and subsequently analyzed using Statistical Package for the Social Sciences (SPSS) software, version 20.0 (IBM Corp., Armonk, NY, USA).

Descriptive statistics

Descriptive statistical analysis was performed to summarize the data. Categorical variables, including demographic characteristics, knowledge-related responses, and attitude and practice patterns, were expressed as frequencies and percentages. These descriptive statistics formed the primary basis for interpretation, in accordance with the exploratory nature of the study.

Inferential statistical analysis

To assess associations between dentists’ knowledge level regarding peri-implantitis and selected demographic variables, inferential statistical tests were applied. Knowledge scores were derived by assigning one point for each correct knowledge-based response, and participants were categorized into adequate and inadequate knowledge groups based on the median score.

The Chi-square test (χ²) was used to evaluate the association between: Knowledge level and years of clinical experience; Knowledge level and primary area of practice (general dentistry vs. specialties).

This test was chosen as the data were categorical and independent. A p-value < 0.05 was considered statistically significant for all inferential analyses.

Knowledge assessment and scoring criteria

Knowledge regarding peri-implantitis was assessed using a subset of knowledge-based questions within the questionnaire that evaluated respondents’ understanding of fundamental concepts related to peri-implant diseases. The knowledge domain included items assessing: Awareness and correct definition of peri-implantitis; Ability to differentiate peri-implant mucositis from peri-implantitis; Identification of common risk factors associated with peri-implantitis; Awareness of diagnostic criteria and clinical signs, such as bleeding on probing, suppuration, increased probing depth, and radiographic bone loss; Knowledge of recommended diagnostic approaches, including clinical examination and radiographic assessment.

For each knowledge-based item, responses were scored using a binary scoring system, where: Correct response = 1; Incorrect or “don’t know” response = 0

Individual knowledge scores were calculated by summing the scores across all knowledge items, yielding a cumulative knowledge score for each participant.

Based on the total knowledge score obtained, respondents were categorized into three knowledge levels as follows: Good knowledge: ≥75% correct responses; Fair knowledge: 50-74% correct responses; Poor knowledge: <50% correct responses

For inferential analysis, including the Chi-square test, knowledge levels were further dichotomized into: Adequate knowledge (Good + Fair); Inadequate knowledge (Poor)

This categorization was adopted to facilitate statistical association testing between knowledge level and practitioner characteristics such as years of clinical experience and specialty of practice.

Risk factor knowledge assessment

Within the knowledge domain, a specific subset of questions assessed respondents’ ability to correctly identify established risk factors for peri-implantitis. Based on current consensus reports and systematic reviews [2,3], the following were considered true risk factors: History of periodontitis; Poor plaque control / inadequate oral hygiene; Smoking; Uncontrolled diabetes mellitus; Lack of regular professional maintenance; Excessive occlusal loading / biomechanical overload

To minimize response bias and assess true conceptual understanding, the questionnaire also included distractor items that are not independently established risk factors for peri-implantitis, including: Patient age alone; Implant brand; Implant length or diameter in isolation; Use of local anesthesia; Gender

Responses were classified as correct only when respondents selected established risk factors and avoided incorrect distractors.

Scoring of risk-related knowledge

Each risk-factor item was scored using a binary system: Correct identification = 1; Incorrect selection or failure to identify = 0.

A risk-related knowledge score was calculated for each participant by summing scores across all risk-factor items.

Based on percentage scores, respondents were categorized as follows: Good risk-factor knowledge: ≥75% correct; Fair risk-factor knowledge: 50-74% correct; Poor risk-factor knowledge: <50% correct.

For inferential analysis, good and fair categories were grouped as adequate risk-factor knowledge, while poor knowledge was considered inadequate.

## Results

A total of 432 responses were initially received. After screening for completeness and eligibility, 26 responses were excluded due to incomplete data or failure to meet the inclusion criteria. The final analysis was therefore conducted on 406 complete and valid responses. Only fully completed questionnaires were included to ensure data consistency, and no imputation was performed for missing responses.

Demographic characteristics

The demographic distribution of the participants is presented in Table 1. The majority of respondents had 1-5 years of clinical experience (68.2%), followed by those with 6-10 years (15.5%), less than 1 year (10.3%), and more than 10 years of experience (5.9%). With respect to the primary area of practice, general dental practitioners constituted the largest group (58.6%). Among specialists, periodontists (13.3%) and prosthodontists (11.3%) formed the predominant subgroups, followed by oral and maxillofacial surgeons (6.9%), conservative dentistry and endodontics specialists (4.9%), orthodontists (3.0%), and pedodontists (2.0%).

**Table 1:** Demographic characteristics of the study participants (n = 406) * : Dental Specialties includes the specialties like Oral Medicine & Radiology, Oral & Maxillofacial Surgery, Oral & Maxillofacial Pathology and Microbiology, Prosthodontics, Periodontology & Implantology, Orthodontics, Conservative Dentistry & Endodontics, Pedodontics & Preventive Dentistry, Public Health Dentistry.

Variable	Category	Frequency (n)	Percentage (%)
Years of Clinical Experience	< 1 year	42	10.3
	1–5 years	277	68.2
	6–10 years	63	15.5
	>10 years	24	5.9
Primary Area of Practice	General Dentistry	238	58.6
	Dental Specialties*	168	41.4

Knowledge regarding peri-implant diseases

Knowledge-related responses are summarized in Table 2. All participants (100%) reported familiarity with the term peri-implantitis. Approximately 65.5% of respondents expressed confidence in their knowledge of peri-implantitis, while 76.1% were able to correctly define the condition. Awareness of diagnostic approaches and common risk factors was reported by 76.1% of participants. Furthermore, 72.2% of the dentists were able to differentiate between peri-implant mucositis and peri-implantitis.

**Table 2:** Knowledge-related responses regarding peri-implant diseases (n = 406)

Knowledge Parameter	Response	Frequency (n)	Percentage (%)
Familiarity with the term “peri-implantitis”	Yes	406	100
Confidence in knowledge of peri-implantitis	Yes	266	65.5
Correct definition of peri-implantitis	Yes	309	76.1
Awareness of diagnostic approaches	Yes	309	76.1
Awareness of common risk factors	Yes	309	76.1
Ability to differentiate peri-implant mucositis from peri-implantitis	Yes	293	72.2
Encounter implant-related cases in practice	Yes	365	89.9
Encounter peri-implantitis cases (frequently/occasionally)	Yes	350	86.1

In terms of clinical exposure, 89.9% of respondents reported encountering implant-related cases in their routine practice, and 86.1% had experienced peri-implantitis cases either frequently or occasionally.

Attitude and clinical practice patterns

Attitude and practice-related responses are detailed in Table 3. Regarding management preferences for peri-implantitis, 47.0% of dentists preferred to refer affected cases to a specialist, while 24.4% reported performing surgical therapy themselves. A smaller proportion (9.4%) relied exclusively on non-surgical treatment approaches.

**Table 3:** Attitude and practice responses toward diagnosis and management of peri-implantitis (n = 406)

Parameter	Response	Frequency (n)	Percentage (%)
Preferred management approach	Referral to specialist	191	47
	Surgical therapy by self	99	24.4
	Non-surgical therapy only	38	9.4
Major challenges in treatment	Patient compliance	304	74.9
	Cost of treatment	258	63.5
Recommended recall interval	Every 3–6 months	184	45.3
	Every 6–12 months	171	42.1
Belief in role of patient education	Significant role	359	88.4
Impact of advances in implant materials/techniques	Some impact	149	36.7
	Significant impact	225	55.4

The most commonly reported challenges in managing peri-implantitis were patient compliance (74.9%) and high treatment costs (63.5%). With respect to maintenance protocols, 45.3% of practitioners recommended recall visits at 3-6 month intervals, whereas 42.1% suggested follow-up intervals of 6-12 months for early detection of peri-implant disease.

A substantial majority of respondents (88.4%) believed that patient education plays a significant role in the prevention of peri-implant mucositis and peri-implantitis. Additionally, 36.7% of dentists perceived that advances in implant materials and techniques have some impact on reducing peri-implant disease risk, while 55.4% believed these advancements have a significant impact.

Inferential statistics

In addition to descriptive findings, inferential analysis revealed a statistically significant association between years of clinical experience and level of knowledge regarding peri-implantitis (Chi-square test, p < 0.05). Dentists with 1-5 years of experience demonstrated a higher proportion of adequate knowledge compared to those with longer clinical experience (Table 4).

**Table 4:** Association between dentist characteristics and knowledge of peri-implantitis Chi-Square Test, *Statistically significant at p < 0.05

Variable	Categories Compared	χ² value	df	p-value
Clinical experience vs Knowledge level	<1 yr, 1–5 yrs, 6–10 yrs, >10 yrs × Adequate / Inadequate	8.72	3	0.033*
Specialty vs Knowledge level	General dentistry vs Specialties × Adequate / Inadequate	10.45	1	0.001*
Experience vs Treatment preference	Experience groups × Treatment choice	4.11	6	0.661

Similarly, specialty of practice showed a significant association with knowledge level (p < 0.05), with periodontists and prosthodontists exhibiting higher levels of knowledge compared to general dental practitioners and other specialties.

No statistically significant association was observed between years of experience and preferred treatment modality (p > 0.05).

**Figure 1:**
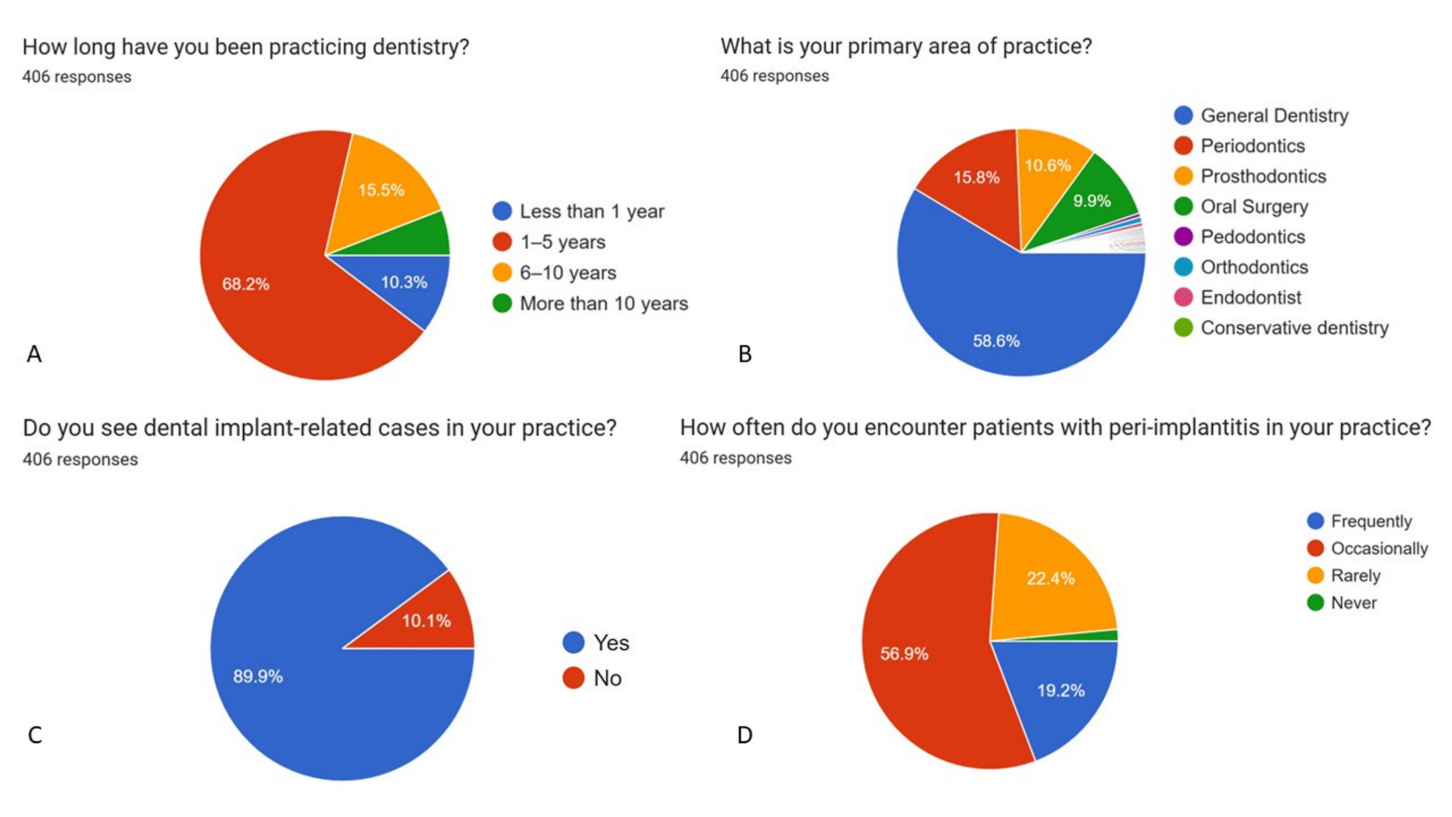
Basic information and basic knowledge of implant dentistry (n = 406) A: How long have you been practicing dentistry? B: What is your primary area of practice? C: Do you see dental implant-related cases in your practice? D: How often do you encounter patients with peri-implantitis in your practice?

**Figure 2:**
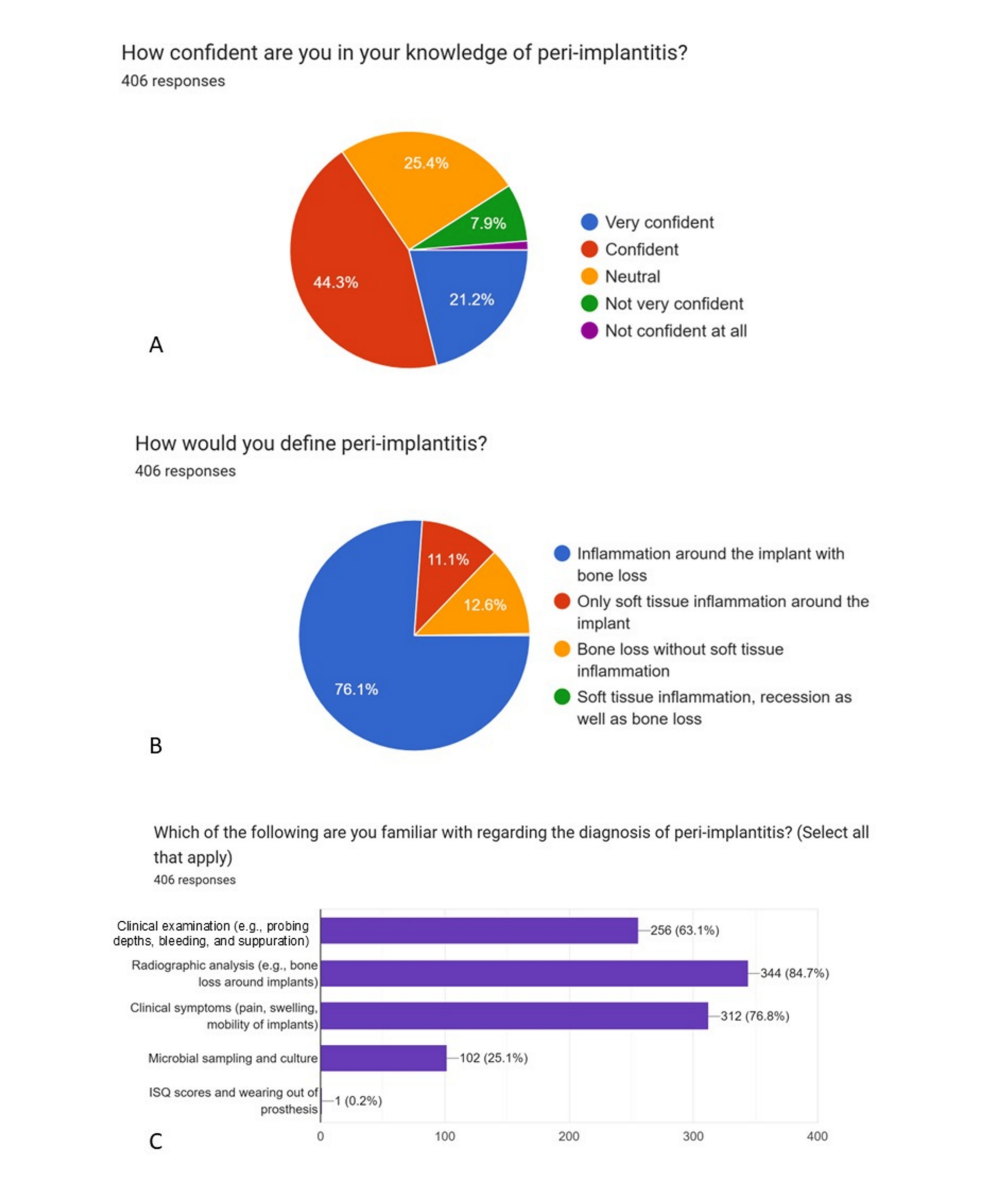
Knowledge of implant-related complications (Part-1) A: How confident are you in your knowledge of peri-implantitis? B: How would you define peri-implantitis? C: Which of the following are you familiar with regarding the diagnosis ofperi-implantitis?

**Figure 3:**
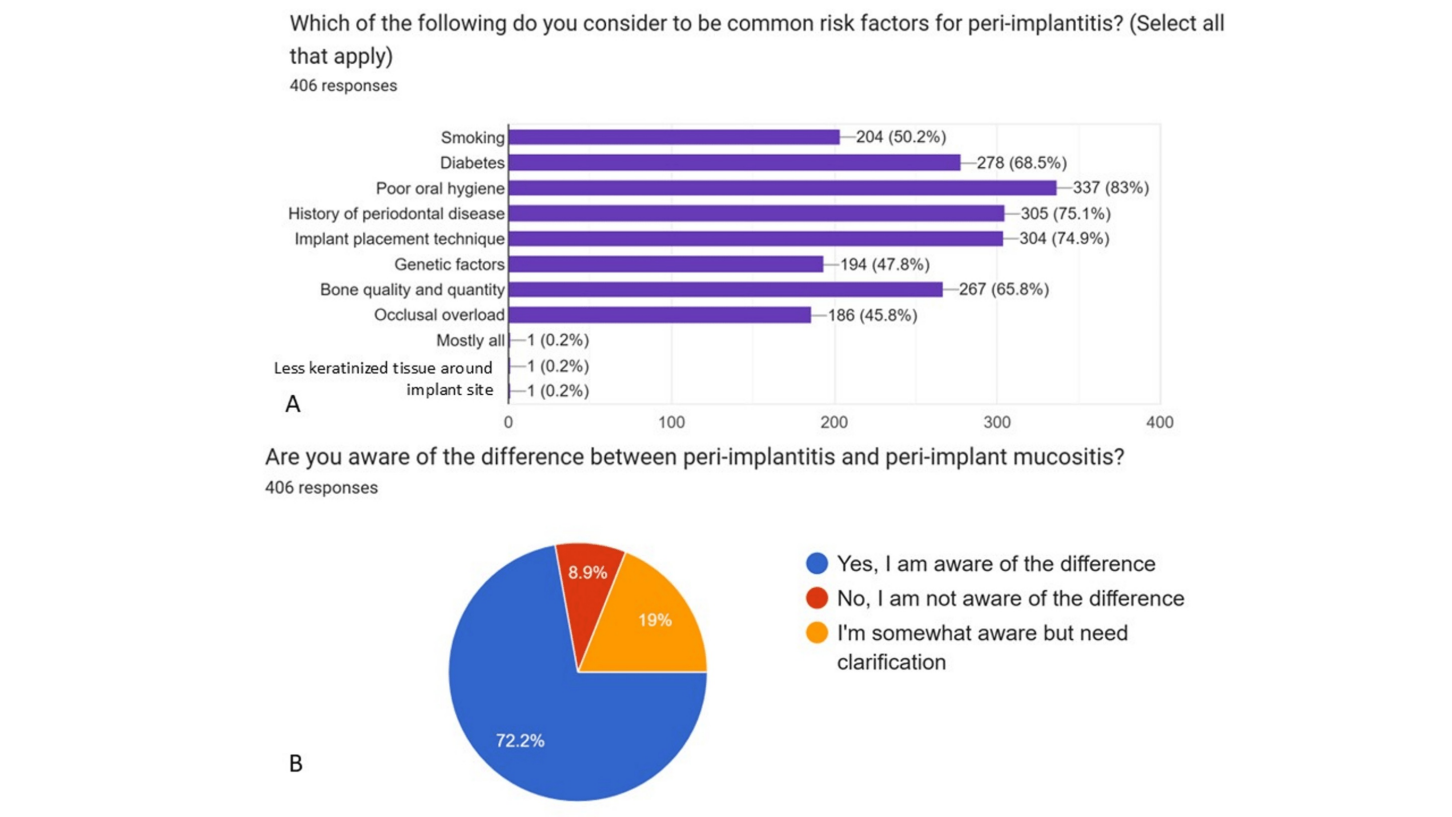
Knowledge of implant-related complications (Part-2) A: Which of the following do you consider to be common risk factors for peri-implantitis? B: Are you aware of the difference between peri-implantitis and peri-implant mucositis?

**Figure 4:**
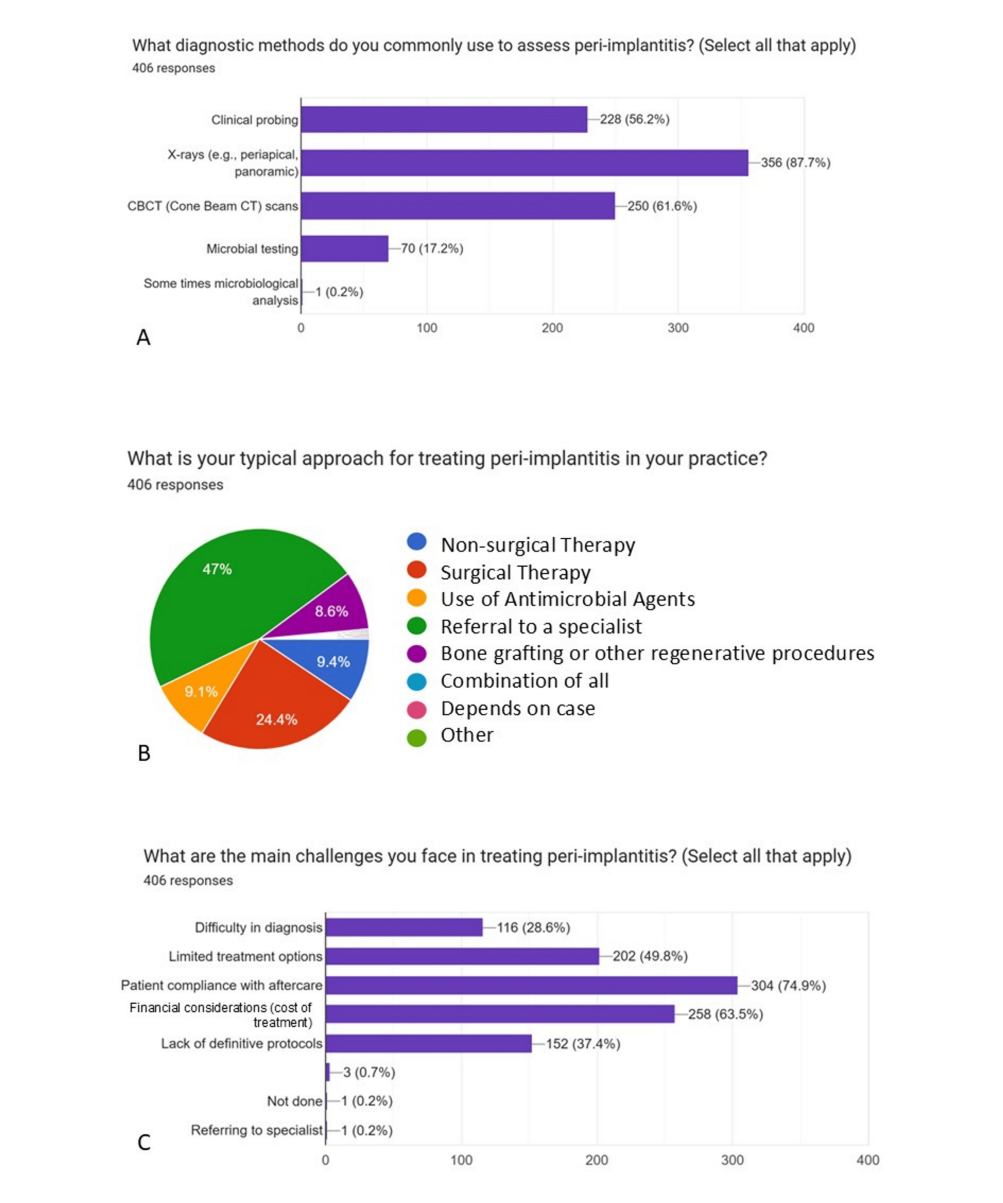
Attitude towards diagnosis & management of implant-related complications (Part-1) A: What diagnostic methods do you commonly use to assess peri-implantitis? B: What is your typical approach for treating peri-implantitis in your practice? C: What are the main challenges you face in treating peri-implantitis?

**Figure 5:**
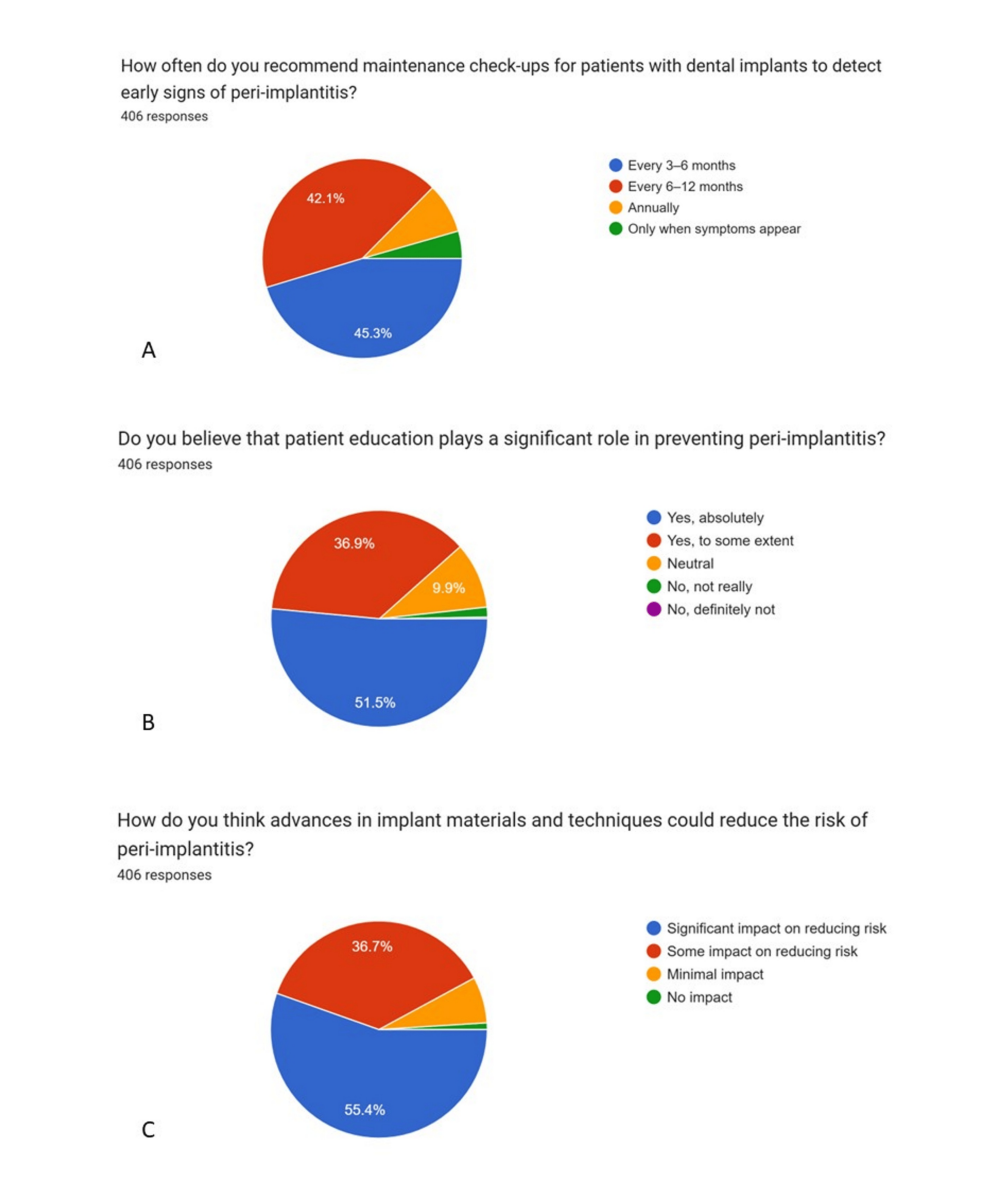
Attitude towards diagnosis & management of implant-related complications (Part-2) A: How often do you recommend maintenance check-ups for patients with dental implants to detect early signs of peri-implantitis? B: Do you believe that patient education plays a significant role in preventing peri-implantitis? C: How do you think advances in implant materials and techniques could reduce the risk of peri-implantitis?

Graphical representations generated using Google Forms (pie charts) (Figures 1-5) have been retained only as supplementary material for visual reference and are not relied upon for primary data interpretation.

## Discussion

The present cross-sectional survey provides valuable insight into the knowledge, attitudes, and clinical practices of dentists in Gujarat State regarding peri-implantitis. Given the increasing reliance on dental implants as a predictable treatment modality with long-term success rates exceeding 90% (Buser et al., 2012) [1], the identification and management of biological complications such as peri-implant diseases have become critically important.

Demographic profile and clinical exposure

A notable finding of this study was the predominance of early-career practitioners, with nearly two-thirds of respondents having 1-5 years of clinical experience. This demographic trend may partially explain the relatively high level of baseline awareness observed, as recent graduates are more likely to have been exposed to updated implant curricula and contemporary diagnostic concepts. Despite differences in years of experience, the high proportion of respondents encountering implant-related cases (89.9%) and peri-implantitis cases (86.1%) highlights the widespread clinical relevance of peri-implant diseases, which demonstrate substantial prevalence at both implant and patient levels.

The diverse specialty representation, with a majority being general dental practitioners, underscores the pivotal role of primary care dentists in implant maintenance and early disease detection. This is particularly relevant in the Indian clinical setting, where access to specialist care may be variable.

Knowledge of peri-implant diseases

Encouragingly, all participants reported familiarity with the term peri-implantitis, and more than three-quarters were able to define it correctly. This reflects improved dissemination of consensus definitions and diagnostic criteria following major workshops and classification updates (Schwarz et al., 2018) [2]. Additionally, 72.2% of respondents were able to differentiate peri-implant mucositis from peri-implantitis, a distinction that is essential for timely intervention, as peri-implant mucositis is considered reversible, whereas peri-implantitis is characterized by progressive bone loss.

However, despite acceptable theoretical awareness, gaps remain in a comprehensive understanding of diagnostic protocols and risk factor assessment. Although 76.1% reported awareness of diagnostic approaches and risk factors, this does not necessarily translate into consistent clinical application, as highlighted in previous consensus reports emphasizing the need for longitudinal assessment and baseline comparisons (Schwarz et al., 2018) [2].

Attitude toward management and referral practices

Management preferences revealed a cautious clinical approach, with nearly half of the respondents preferring to refer peri-implantitis cases to specialists. This finding reflects appropriate clinical judgment, considering the complexity and unpredictable outcomes associated with peri-implantitis therapy. Systematic reviews have consistently demonstrated that non-surgical therapy alone is often insufficient in managing established peri-implantitis, particularly in advanced cases (Renvert & Polyzois, 2015) [5].

Nonetheless, the fact that nearly one-quarter of dentists reported performing surgical therapy independently and 9.4% relied exclusively on non-surgical approaches highlights variability in treatment strategies. This heterogeneity mirrors findings from previous literature and underscores the absence of universally standardized treatment protocols, despite extensive consensus efforts.

Challenges in clinical practice

Patient compliance emerged as the most significant challenge in peri-implantitis management, followed by treatment cost. These barriers are well-documented in implant literature and significantly influence treatment outcomes. Anti-infective preventive strategies and long-term maintenance programs have been shown to reduce the incidence of biological complications, yet their success is highly dependent on patient adherence (Salvi & Zitzmann, 2014; Heitz-Mayfield et al., 2014) [6,7].

The strong emphasis placed by respondents on patient education (88.4%) aligns with evidence indicating that poor plaque control is a major etiological factor in peri-implant disease development (Sanz et al., 2012; Lindhe & Meyle, 2008) [8,9]. This finding reflects growing awareness among practitioners regarding the preventive aspect of implant care.

Maintenance protocols and technological advances

The majority of respondents recommended recall intervals ranging from 3 to 12 months, with nearly half favoring 3-6 month follow-ups. This approach is consistent with consensus guidelines advocating individualized maintenance schedules based on patient risk profiles (Berglundh et al., 2018; Sanz et al., 2020) [10,11]. Regular monitoring is essential, given that peri-implant diseases often progress silently until advanced tissue destruction occurs.

More than half of the participants believed that advances in implant materials and techniques significantly reduce the risk of peri-implant diseases. While innovations in implant surface characteristics and prosthetic designs may contribute to improved peri-implant tissue responses, consensus reports emphasize that technology alone cannot compensate for inadequate diagnosis, surgical precision, or maintenance care (Berglundh et al., 2018) [10].

Integration of inferential results

The inferential analysis further strengthens the findings of the present study by demonstrating statistically significant associations between dentists’ knowledge levels and both clinical experience and specialty. The observed association between years of experience and knowledge suggests that cumulative clinical exposure and continued engagement with implant-related cases may enhance familiarity with peri-implant disease diagnosis and management. Conversely, it also reflects the benefit of recent academic exposure among early-career practitioners, as contemporary curricula increasingly emphasize implant maintenance and biological complications.

The significant association between specialty and knowledge level is clinically relevant. Specialists-particularly periodontists and oral surgeons-are more likely to receive structured training in peri-implant tissue biology, risk assessment, and evidence-based management protocols. This finding is consistent with previous reports indicating superior awareness and confidence among specialists compared with general practitioners in managing peri-implant diseases [5,7].

These associations underscore the importance of targeted continuing dental education (CDE) programs for general practitioners, focusing on early diagnosis, risk factor modification, and appropriate referral protocols. While implant therapy has become increasingly common in general practice, the management of peri-implantitis remains complex and technique-sensitive, warranting either advanced training or timely referral to specialists.

Importantly, although statistically significant associations were identified, the cross-sectional nature of the study precludes causal inference. Nonetheless, the findings highlight meaningful trends that can inform educational strategies and reinforce the need for standardized guidelines in implant maintenance across all dental disciplines.

Overall interpretation

When combined, the study's findings indicate that although Gujarati dentists have a basic understanding of peri-implantitis, there are still significant gaps in their clinical approach, particularly with relation to referral and treatment plans. These findings mirror those of a study by Jain et al. (2021) [12], which reported that although most dentists are aware of peri-implant diseases, fewer are confident in diagnosing and treating them effectively.

The findings from this study highlight critical deficiencies in the understanding and management of peri-implantitis among dentists in Gujarat. While awareness of peri-implantitis as a disease was relatively high, in-depth knowledge of diagnostic criteria, risk factor interplay, and management strategies was lacking, consistent with similar studies in both Indian and international contexts.

In a study conducted by Al-Aali et al. (2019) [13], several doctors expressed hesitancy over the use of probing around implants, suggesting a more general hesitancy or lack of training in peri-implant assessment methods.

The underutilization of probing and baseline radiographic comparison in this study mirrors these global trends and suggests a widespread gap in translating academic knowledge into clinical practice. The reluctance to probe may stem from outdated fears of damaging the peri-implant seal, an idea disproven by studies showing that gentle probing with plastic or titanium instruments is both safe and diagnostically critical (Klinge et al., 2015) [14].

Another major concern revealed was the lack of awareness of systemic and biomechanical risk factors. The link between diabetes and peri-implant bone loss is well-documented. Diabetes mellitus/hyperglycaemia is known to be associated with a greater risk of peri-implantitis. Similarly, excessive occlusal loading from bruxism or poor prosthesis design exacerbates peri-implant inflammation, yet less than half of respondents considered these factors.

This limited recognition may partly result from a compartmentalized view of implantology within general practice, where broader systemic assessments are underemphasized. Enhancing interdisciplinary understanding, especially regarding periodontics and systemic health, is therefore essential.

Although general awareness of peri-implantitis was high among respondents, the present study revealed important discrepancies in risk-factor-specific knowledge, particularly regarding systemic and biomechanical contributors. This finding is clinically relevant, as failure to recognize these modifiers may delay early diagnosis and compromise preventive strategies. Similar observations have been reported in previous surveys, where dentists demonstrated stronger familiarity with plaque-related risk factors but limited understanding of systemic and occlusal influences [6,9,12]. These results underscore the importance of reinforcing risk-based assessment models in implant maintenance protocols.

The lack of continuing education participation is also noteworthy. While time constraints and cost are common barriers, the digital transformation of dental education offers new opportunities. Webinars, e-learning modules, and interactive case discussions can bridge knowledge gaps cost-effectively.

From a public health standpoint, this study underscores a critical need for capacity-building among general dentists. Dentists often serve as the primary care providers in dental settings and must be adequately equipped to identify peri-implant disease at its earliest stages.

This study has certain limitations that should be acknowledged. The absence of formal reliability testing represents a limitation of the study and should be considered when interpreting the findings. The use of convenience sampling and the absence of district-wise stratification may limit the generalizability of the findings across the entire state, and may have introduced selection bias, limiting the generalizability of findings beyond the surveyed region of Gujarat. As a cross-sectional, self-reported survey, the results reflect participants’ perceived rather than objectively measured knowledge, making them susceptible to recall and social desirability bias. Although the questionnaire was validated, the structured response format restricted exploration of more complex clinical opinions. Additionally, the study did not correlate practitioners’ knowledge with actual clinical performance or patient outcomes, and the relatively lower representation of specialists may have influenced the interpretation of management practices. Despite these constraints, the findings provide valuable preliminary insights into existing awareness gaps and emphasize the need for broader, multicentric studies integrating objective clinical evaluations.

Ultimately, peri-implantitis is a preventable and manageable condition, but only with the application of evidence-based practices. As new implant surfaces, biomaterials, and diagnostic tools emerge, practitioner training must evolve in parallel.

## Conclusions

A serious risk to the long-term viability of dental implants is peri-implantitis. This survey of dentists in Gujarat reveals that although general awareness of peri-implantitis exists, there is a pronounced gap in the detailed understanding of its risk factors, diagnosis, and evidence-based management. This discrepancy emphasizes the urgent need for continuing education and clinical guidelines to be disseminated and implemented at the primary care level.

Bridging these knowledge gaps through targeted training programs, interdisciplinary collaboration, and integration of peri-implant assessment into routine care can enhance implant outcomes and ensure long-term patient satisfaction.

## Appendices

Assessment of knowledge regarding risk of peri-implantitis after dental implant placement among Dental Practitioners of Gujarat State: A questionnaire survey

      * Indicates required question       

Basic information

1. How long have you been practicing dentistry? *

(Mark only one)

Less than 1 year  

1-5 years

6-10 years

More than 10 years

2. What is your primary area of practice? *

(Mark only one)

General Dentistry

Periodontics

Prosthodontics

Oral Surgery

Other: __________________________                                                       

Basic knowledge of Implant dentistry

3. Do you see dental implant-related cases in your practice? *

(Mark only one)

Yes  

No

4. How often do you encounter patients with peri-implantitis in your practice? *

(Mark only one)

Frequently  

Occasionally  

Rarely

Never

Knowledge of Implant-related complications

5. How confident are you in your knowledge of peri-implantitis? *

(Mark only one)

Very confident  

Confident  

Neutral

Not very confident  

Not confident at all

6. How would you define peri-implantitis? *

(Mark only one)

Inflammation around the implant with bone loss

Only soft tissue inflammation around the implant

Bone loss without soft tissue inflammation

Other: _________________________

7. Which of the following are you familiar with regarding the diagnosis of peri-implantitis? *

(Select all that apply)

Clinical examination (e.g., probing depths, bleeding, and suppuration)  

Radiographic analysis (e.g., bone loss around implants)

Clinical symptoms (pain, swelling, mobility of implants)  

Microbial sampling and culture

Other: ____________________________________                                                                        

8. Which of the following do you consider to be common risk factors for peri-implantitis? *

(Select all that apply)

Smoking  Diabetes

Poor oral hygiene

History of periodontal disease  

Implant placement technique  

Genetic factors

Bone quality and quantity  

Occlusal overload

Other: _________________________________

9. Are you aware of the difference between peri-implantitis and peri-implant mucositis? *

(Mark only one)

 Yes, I am aware of the difference  

No, I am not aware of the difference

I'm somewhat aware but need clarification

Attitude towards diagnosis & management of Implant-related complications

10. What diagnostic methods do you commonly use to assess peri-implantitis? *

(Select all that apply)

Clinical probing

X-rays (e.g., periapical, panoramic)  

CBCT (Cone Beam CT) scans

Microbial testing

Other: __________________________________

11. What is your typical approach for treating peri-implantitis in your practice? *

(Mark only one)

Non-surgical therapy (scaling and root planing)

Surgical therapy (flap surgery and debridement)

Use of antimicrobial agents

Referral to a specialist (periodontist, oral surgeon)

Bone grafting or other regenerative procedures

Other: ______________________________                                                  

12. What are the main challenges you face in treating peri-implantitis? *

(Select all that apply)

Difficulty in diagnosis

Limited treatment options

Patient compliance with aftercare

Financial considerations (cost of treatment)  Lack of definitive protocols

Other: ___________________________________

13. How often do you recommend maintenance check-ups for patients with dental implants to detect early signs of peri-implantitis? *

(Mark only one)

Every 3-6 months

Every 6-12 months  Annually

Only when symptoms appear

14. Do you believe that patient education plays a significant role in preventing peri-implantitis? *

(Mark only one)

Yes, absolutely  

Yes, to some extent  

Neutral

No, not really  

No, definitely not

15. How do you think advances in implant materials and techniques could reduce the risk of peri-implantitis? *

(Mark only one)

Significant impact on reducing risk  

Some impact on reducing risk  

Minimal impact

No impact

## References

[1] Buser D, Janner SF, Wittneben JG, Brägger U, Ramseier CA, Salvi GE (2012). 10-year survival and success rates of 511 titanium implants with a sandblasted and acid-etched surface: A retrospective study in 303 partially edentulous patients. Clin Implant Dent Relat Res.

[2] Schwarz F, Derks J, Monje A, Wang HL (2018). Peri-implantitis. J Clin Periodontol.

[3] Derks J, Tomasi C (2015). Peri-implant health and disease. A systematic review of current epidemiology. J Clin Periodontol.

[4] Diaz P, Gonzalo E, Villagra LJ, Miegimolle B, Suarez MJ (2022). What is the prevalence of peri-implantitis? A systematic review and meta-analysis. BMC Oral Health.

[5] Renvert S, Polyzois IN (2015). Clinical approaches to treat peri-implant mucositis and peri-implantitis. Periodontol 2000.

[6] Salvi GE, Zitzmann NU (2014). The effects of anti-infective preventive measures on the occurrence of biologic implant complications and implant loss: A systematic review. Int J Oral Maxillofac Implants.

[7] Heitz-Mayfield LJ, Mombelli A (2014). The therapy of peri-implantitis: A systematic review. Int J Oral Maxillofac Implants.

[8] Sanz M, Chapple IL (2012). Clinical research on peri-implant diseases: Consensus report of Working Group 4. J Clin Periodontol.

[9] Lindhe J, Meyle J (2008). Peri-implant diseases: Consensus report of the Sixth European workshop on periodontology. J Clin Periodontol.

[10] Berglundh T, Armitage G, Araujo MG (2018). Peri-implant diseases and conditions: Consensus report of workgroup 4 of the 2017 world workshop on the classification of periodontal and Pperi-implant diseases and conditions. J Clin Periodontol.

[11] Sanz M, Herrera D, Kebschull M (2020). Treatment of stage I-III periodontitis-The EFP S3 level clinical practice guideline. J Clin Periodontol.

[12] Jain M, Bin Shaffa'ee MS, Uppoor A, Pralhad S, Nayak SU, Saldanha S (2022). Evaluation of risk factors of peri-implant disease using a new manual risk assessment model: A clinical study. Int J Dent.

[13] Al-Aali KA, Alharbi AA (2019). Peri-implantitis knowledge among dental professionals. Saudi Dent J.

[14] Klinge B, Meyle J (2012). Peri-implant tissue destruction. The Third EAO consensus conference 2012. Clin Oral Implants Res.

